# Quality of Life and Its Association with Effort-Reward Imbalance among Medical Officers Working in Government Hospitals in Kuching, Sarawak, Malaysia

**DOI:** 10.21315/mjms2022.29.5.11

**Published:** 2022-10-28

**Authors:** Chin Lie Joo, Maruzairi Husain, Nor Asyikin Fadzil, Yee Cheng Kueh

**Affiliations:** 1 Department of Psychiatry, Hospital Umum Sarawak, Sarawak, Malaysia; 2 Department of Psychiatry, School of Medical Sciences, Universiti Sains Malaysia, Kelantan, Malaysia; 3 Unit of Biostatistics and Research Methodology, School of Medical Sciences, Universiti Sains Malaysia, Kelantan, Malaysia

**Keywords:** physicians, quality of life, effort-reward imbalance, occupational stress, Malaysia

## Abstract

**Background:**

Medical officers (MOs) face multiple sources of work-related stress, including work system transition, job insecurity, dissatisfaction with income and intense working environments. This study aimed to examine the quality of life (QOL), effort-reward imbalance (ERI) and their association among MOs working in government hospitals in Kuching, Sarawak, Malaysia.

**Methods:**

This cross-sectional study was conducted among MOs from Sarawak General Hospital and Hospital Sentosa from May 2018 to March 2020. A total of 614 participants were selected through convenient sampling. An email with a link to three sets of questionnaires via Google forms including a questionnaire on sociodemographic data and job characteristics, the World Health Organization Quality of Life-Brief version (WHOQOL-BREF) (Malay version) and the effort-reward imbalance (ERI-Q) (long version) was sent to potential participants. A total of 276 MOs completed and returned the questionnaires. Data were analysed using descriptive, simple and multiple logistic regression analysis. A *P*-value of less than 0.05 was taken as a statistically significant result.

**Results:**

Most MOs reported no adversity in the workplace (i.e. 29% low effort/high reward, 5.1% high effort/high reward, 6.2% low effort/low reward and 23.6% high effort/low reward). More than half of MOs (54%) reported poor general QOL and were associated with a combination of active and passive on-calls (adjusted odds ratio [AOR] = 5.36, 95% confidence interval [CI]: 1.21, 23.79). Poor QOL in the physical domain was associated with the presence of chronic illness (AOR = 23.35; 95% CI: 4.25, 128.45), active on-calls (AOR = 14.75; 95% CI: 1.16, 188.35) and a combination of active and passive on-calls (AOR = 18.25; 95% CI: 1.39, 238.98). Men had a higher risk of poor QOL in the environmental domain (AOR = 2.03; 95% CI: 1.04, 3.98). Only 23.6% of MOs reported psychosocial adversity at work (high effort/low reward). High effort/low reward was associated with poor QOL in general (AOR = 4.71; 95% CI: 1.71, 13.01), physical (AOR = 4.53; 95% CI: 2.02, 10.17), psychological (AOR = 5.95; 95% CI: 2.82, 12.58) and environmental domains (AOR = 4.21; 95% CI: 1.95, 9.08). Low effort/high reward was found to have a lower likelihood of poor QOL in the social domain (AOR = 0.13; 95% CI: 0.04, 0.44).

**Conclusion:**

Higher ERI was found to be associated with poor QOL among MOs in government hospitals. Future research should focus on interventions to improve working conditions.

## Introduction

The health system in Malaysia traditionally divides doctors into house officers (HOs) (i.e. doctors undergoing internship training with a provisional practice certificate) (1), medical officers (MOs) (i.e. doctors who completed an internship with a full practice certificate) (2) and specialists (i.e. doctors who completed specialist training and are registered with National Specialist Registrar) (2). MOs are shouldered with patient care as well as multiple challenges, including work system transition (i.e. shift system to on-call system along with the transition from HOs to MOs), job insecurity and difficulties in career advancement (3). The increased supply of medical graduates has caused saturation in the availability of positions in the workforce and opportunities for career progression (4). This is complicated further by the fact that the Malaysian government introduced a contract basis system for HOs and a policy whereby permanent appointment as an MO depends on performance and vacancy of position (5). Therefore, an increase in job stress is anticipated which will likely negatively impact the quality of life (QOL) (6). Moreover, doctors with poor QOL will in turn affect the quality of patient care (7).

According to the World Health Organization (WHO), QOL is defined as ‘an individual’s perception of their position in life in the context of the culture and value systems in which they live and in relation to their goals, expectations, standards and concerns’ (8). Assessment of QOL consists of the perception of general QOL, perception of general health and four domains: i) physical, ii) psychological, iii) social and iv) environmental domains (Table 1) (8). Work is an integral part of the social domain that can impact QOL. Studies have shown that non-conducive working environments were associated with physical disorders (9, 10), psychological disorders (11) and poor QOL (6). Flexibility at work has been shown to improve workers’ general well-being and health (12).

Effort-reward imbalance (ERI) is a theoretical model used in the study of occupational health. The model focuses on the reciprocity of exchange between the effort spent and the reward gained at work (13). Effort is further divided into extrinsic and intrinsic efforts. Extrinsic effort refers to the physical or psychological demands of a job (e.g. workload, responsibilities and time spent on the job) (13), while intrinsic effort refers to the personal and inner motivation of an employee (13). Overcommitment is a set of behaviours and emotions with a combination of excessive striving and a strong desire for approval and esteem (13). According to the ERI model, overcommitted individuals are noted to be at higher risk of strain, hence, becoming more detrimental to health (13). Meanwhile, reward can also be divided in the form of money (e.g. salary or allowance), esteem (e.g. respect and support from colleagues or superiors) and job security (e.g. career advancement, employment status or promotion) (13). Thus, ERI is produced by combining effort and reward. For example, a worker with short working hours, low workload and high salary is an example of low effort/high reward. ERI is generally divided into four categories: i) low effort/high reward (LEHR), ii) high effort/high reward (HEHR), iii) low effort/low reward (LELR) and iv) high effort/low reward (HELR) (7). There is increasing utilisation of the ERI model in the study of occupational health (13), including a study conducted among academicians in Malaysia (14).

ERI has been shown to affect different domains in QOL. Studies have shown that ERI and overcommitted workers experience more negative effects on their health, including high blood pressure (15), increased serum cortisol (15), alcohol use (16), absence from work due to illness (17) and psychological wellbeing (18). A study conducted among automobile workers in Malaysia showed high job demand was associated with lower scores in the environmental domain (6). This was supported by another study that revealed poorer QOL in the environmental domain in those with HELR (7). Teles et al. (7) also found that those with low reward had a higher prevalence of lower scores in the social domain.

According to the Malaysian Medical Association, an estimated 300–400 doctors resign from government services yearly due to high workload, dissatisfaction with income and working environment (19). However, compared to HOs, issues involving MOs were discussed less frequently (20). Most local studies conducted exclusively on MOs (1, 21–23) focused on the level of fatigue and recovery, psychological health and job satisfaction, but not on the holistic aspect of QOL. The identification of key factors that affect ERI and QOL may provide a means to ensure well-motivated staff who can provide a better quality of care.

To date, there is no study on QOL and its association among MOs working in government hospitals in Malaysia. This study aimed to assess QOL and its association with ERI among MOs working in government hospitals in Kuching, Sarawak. This study also examined the relationship between sociodemographics, job characteristics and QOL. We hypothesised that MOs exposed to HELR at the workplace are associated with a higher risk of poor QOL.

## Methods

### Design and Participants

This was a cross-sectional study conducted among MOs at Sarawak General Hospital (SGH) and Hospital Sentosa in Sarawak from May 2018 to March 2020. A sample size of 614 participants was calculated using the Power and Sample Size Program with type 1 error set at 0.05 and power set at 0.8, according to a study conducted among healthcare workers in Brazil with a 40% of dropout rate (14). A list of email addresses was collected from both hospitals between the periods May 2018 to June 2018 and June 2019 to March 2020. Duplicate email addresses collected during the second period were discarded. A total of 614 email addresses were collected from SGH and Hospital Sentosa via convenient sampling (i.e. 250 from the first period and 364 from the second period). An email with a link to three sets of questionnaires via Google forms including a questionnaire on sociodemographic data and job characteristics, the World Health Organization Quality of Life-Brief version questionnaire (WHOQOL-BREF) (Malay version) and the effort-reward imbalance questionnaire (ERI-Q) (long version) as well as research subject information was sent to the selected participants. Inclusion criteria were MOs that worked in either SGH or Hospital Sentosa. HOs and specialists were excluded from the study. Those who did not respond within one month’s time were encouraged to participate via a verbal reminder and a second email. Non-responders were defined as those who did not submit questionnaires by the end of March 2020. Of 614 potential participants, only 276 completed the questionnaires with a response rate of 45%.

### Instruments

Participants were asked to provide information on their sociodemographic data, including age, gender, race, religion, marital status, number of children and any chronic medical or psychiatric illnesses. Job characteristics of participants included the duration of service as an MO, current department, involvement in postgraduate studies, contract or permanent MO status and types of working hours or on-calls.

The WHOQOL-BREF (Malay version) was used to measure QOL. WHOQOL-BREF is a self-administered questionnaire with 26 items and each item is rated by a 5-point Likert interval scale (8). A higher score indicates better QOL (8), while poor QOL is defined as any score that falls within the lowest quartile (7) in each domain. The psychometric study of WHOQOL-BREF (Malay version) was noted to have satisfactory internal consistency. The Cronbach’s alpha for all four domains were as follows: physical (0.80), psychological (0.64), social relationship (0.65) and environment (0.73) (25). The Cronbach’s alpha also showed good test-retest reliability (24).

ERI was assessed with the ERI-Q long version based on the ERI model. The ERI-Q is divided into three categories: i) effort (6 items), ii) reward (10 items) and iii) overcommitment (6 items) (26). It is a self-administered questionnaire and all items are rated with a 4-point Likert rating scale. Generally, a higher score in each category equals higher effort, higher reward or higher overcommitment (25). The total sum of the score for each category (effort, reward and overcommitment) that falls into the highest tertile is considered to be high effort, reward or overcommitment (7) and vice versa. The ERI-Q showed satisfactory internal consistency with a Cronbach’s alpha of more than 0.7 for all categories (25), with acceptable test-retest reliability (26).

### Analysis of Data

The data entry and analysis were performed using Statistical Package for the Social Sciences (SPSS) version 24. Descriptive statistics were used for the sociodemographic and job characteristics of participants. Numerical data were not normally distributed; hence, data were presented as median (interquartile range [IQR]). Categorical data were presented as frequency (percentage). Simple logistic regression was used to analyse the association between independent variables with general QOL and the domains of QOL to get the crude odds ratio, 95% confidence interval (95% CI) and statistical significance (*P*-value). Variables that achieved a *P*-value of less than 0.25 and theoretically important variables were included in the multiple logistic regression analysis. Model fitness was checked with Hosmer-Lemeshow goodness of fit. A *P*-value of more than 0.05 signified the model is fit. A *P*-value of less than 0.05 was taken as a statistically significant result.

## Results

A total of 276 MOs participated in the study with a 45% of response rate. The median age of participants was 30 (IQR = 3) years old. Among the 276 participants, 161 (58.3%) were female and 115 (41.7%) were male. The majority were Chinese (63.4%), single (65.6%) and without any medical or psychiatric illness (92.4%). Medical illnesses elicited in this study included hypertension, dyslipidemia, bronchial asthma, eczema, spine disease (prolapse intervertebral disc and ankylosing spondylitis), Meniere’s disease, endometriosis, migraine, kidney disease and thyroid disorder. Only one participant reported having major depressive disorder. Most participants reported no children (81.2%). The average duration of service for an MO was 3.5 (IQR = 2.5) years. Most were involved in postgraduate studies (51.8%) and were permanently employed (98.9%). In general, most MOs were required to work active on-calls (37.7%) and a combination of active and passive on-calls (34.3%) (Table 2).

The effort-reward assessment of psychosocial working conditions found that most MOs reported no adversity in the workplace, represented by 29% LEHR, 5.1% HEHR, 6.2% LELR and 36.2% reported neither high nor low in effort-reward. Furthermore, 23.6% of MOs reported HELR and 38.4% reported high overcommitment. More than half of MOs (54%) reported poor general QOL. The results also showed that 44.6% of MOs perceived having poor health. The prevalence of poor QOL for each domain was as follows: 29% in the physical and psychological domain, 30.4% in the social domain and 27.5% in the environmental domain.

MOs who worked both active and passive on-calls were found to have around five times the odds of experiencing poor general QOL compared to those who worked during regular office hours. HELR was found to be associated with a higher risk of poor general QOL and vice versa for the LEHR group compared to those who reported medium effort and reward (Table 3).

A higher likelihood of poor QOL in the physical domain was associated with the presence of chronic illness, active on-calls, and a combination of active and passive on-calls (Table 4). MOs with LEHR were less likely to report poor QOL in physical and psychological domains and the opposite was reported in those with HELR (Table 4). As for the social domain, a lower risk of poor QOL was reported in MOs who had LEHR (Table 5). Men were more likely to report poor QOL in the environmental domain compared to women. The likelihood of poor QOL in the environmental domain in MOs with HELR was higher, which was approximately four times the odds compared to those with medium effort-reward. In contrast, MOs with LEHR had around 96% reduced odds of experiencing poor QOL in the environmental domain compared to those with medium effort-reward (Table 5).

## Discussion

To our knowledge, this is the first study on QOL and ERI among MOs in Malaysia. Overall, this study supported our hypothesis in that MOs who experienced HELR at the workplace had a higher risk of experiencing poor QOL. Our study revealed a similar association with a previous study that reported the presence of chronic medical or psychiatric illness was associated with a higher chance of poor QOL in the physical domain (7). Both studies in Brazil and Taiwan among healthcare workers revealed a similar result in the association between HELR with poor QOL in general, physical, psychological and environmental domains (7, 18). This is in line with our findings that MOs who reported LEHR were more likely to be protected from poor QOL in the general, physical, psychological and social domains.

More than half of MOs (54%) reported poor general QOL, which was significantly higher compared to primary healthcare workers in Brazil (7). This might have been due to the differences in the studied population, culture and occupational settings. Our study noted discrepancies wherein most MOs did not report HELR, but more than half reported poor general QOL. This suggests that there might be an interplay of other factors, such as personality with poor coping skills (27), life conflicts, life events or social support (outside of work-life), which were not assessed in this study. Active on-call duty and a combination of active and passive on-call duties were associated with a higher likelihood of poor QOL in the general and physical domains. It was not surprising that on-call duty was one of the most stressful components in doctors’ work life as it consists of an element of uncertainty, longer working hours and interruptions to family and social life (28). Studies have also shown that long working hours were associated with poor QOL (29), higher stress levels, higher risk of injury at work, lower cognitive function and poorer physical health (30).

This study found that LEHR was associated with a lower risk of poor QOL in the social domain, whereby, the previous study revealed LELR was associated with a higher prevalence of poor QOL in the social domain (7). The key difference lies in the level of reward. Hence, we suggested that reward can be a determining factor in the QOL of the social domain. Being in control at the workplace has a direct association with social relationships (6). Lack of respect or support at the workplace with inequality of job promotion will inversely affect social relationships in life either within or outside work (7).

Male MOs were found to experience a higher risk of poor QOL in the environmental domain. A study conducted among men in Asian countries (China, Japan, Korea, Malaysia and Taiwan) revealed that jobs, honour and control of life are attributed to masculinity (31). Men in Malaysia perceived wealth and jobs as the most important attributes in the definition of masculinity (31, 32). Therefore, perception of masculinity correlates with poor QOL in the environmental domain, which could be due to the presence of dissatisfaction with work and income (7). MOs with HELR had a higher likelihood of poor QOL in the environmental domain (7).

In addition, our study found that those with LEHR were associated with a lower risk of poor QOL in the environmental domain. This reflected that high workload and job strain along with incompatible salary, job security, career advancement and unhealthy working environments led to the perception of financial instability, unhealthy surroundings, lack of opportunities in gaining information or skills and reduced recreational activities (7). Ramlan et al. (19) described that doctors were more satisfied with a high reward at work, including esteem gained from work, good career prospects, support from colleagues and flexible working hours.

### Limitations and Recommendations

One of the limitations of this study was the poor response rate (45%), leading to a failure to achieve the calculated sample size. This rate corresponded to another study among healthcare workers, which found that physicians had the lowest response rate (37.5%) compared to nurses (67.5%) and other hospital staff (62.9%) (e.g. administrative personnel, pharmacists, social workers, psychologists and laboratory workers) (18). The poor response rate in the online surveys may have been due to the lack of updated email addresses and/or owning multiple email addresses (33). Non-response bias may have occurred due to poor response rates as well. Statistical power may be reduced due to a small comparison group. This might be the reason for some significant results with a wide 95% confidence interval.

Poor response rate, convenient sampling and only recruiting MOs from a tertiary hospital in Kuching (capital city of Sarawak) may have limited generalisation and may not represent the true general population. Therefore, future studies can improve by using random sampling, expanded study sites and increased response rates. The response rate may be improved by obtaining updated email addresses, offering incentives and promoting awareness of occupational health. Like the previous study (14), we were not able to rule out reverse causation, as the independent and dependent variables were closely related.

Information bias may have occurred as this study relied on self-reported questionnaires from individual participants, which may have caused underestimation or overestimation of the outcome. Measures taken to minimise information bias included providing participants with research subject information via email, assurance on confidentiality whereby information would not be revealed publicly (unless disclosure was required by law) with no personal identification. Recall bias was minimised in this study as most of the questions were about the current work situation, except for questions in the WHOQOL-BREF that enquired about participants’ condition for the past two weeks.

Contract MOs were found to be more vulnerable to poor QOL (19). Unfortunately, our study failed to recruit adequate contract MOs to study the relationship between workplace adversity and QOL in this group. Lack of responses from contract MOs may have been due to their concern about risking their career advancement. This shortfall may be improved in future studies by promoting occupational health and assurance of confidentiality.

Recommendations for future research include research not only on ERI and QOL, but also on their effect on work performance, quality of care to patients and psychological well-being among MOs. The importance of the impact of working conditions was supported by a study conducted among hospital doctors in Ireland, which focused on the enhancement of working conditions with the statement ‘whether it is acceptable to continue to expect doctors to perform well within a system that demands so much but provides so little support’ (34). Future research that studies the intervention in improving working conditions will also provide policymakers with more insight into occupational health among MOs, which would benefit both doctors and patients’ care. A prospective cohort study design may be used for future studies to establish a causal relationship between ERI and QOL.

## Conclusion

In conclusion, higher ERI (i.e. HELR) was found to be associated with poor QOL among MOs in government hospitals. Policymakers should consider the impact of psychosocial working conditions on the QOL among MOs, as it may affect their job performance and quality of patient care. Policymakers may also focus on either reducing the effort or increasing the reward depending on the availability of resources. Future research should also focus on evidence-based intervention to improve working conditions, which may serve as guidance for policymakers in the planning of future policy.

## Figures and Tables

**Table 1:** WHOQOL-BREF domains

Domains	Facets incorporated within domains
Physical health	Activities of daily living Dependence on medicinal substances and medical aids Energy and fatigue Mobility Pain and discomfort Sleep and rest Work capacity
Psychological	Bodily image and appearance Negative feelings Positive feelings Self-esteem Spirituality/religion/personal beliefs Thinking, learning, memory and concentration
Social	Personal relationships Social support Sexual activity
Environmental	Financial resources Freedom, physical safety and security Health and social care: accessibility and quality Home environment Opportunities for acquiring new information and skills Participation in and opportunities for recreation/leisure activities Physical environment (pollution/noise/traffic/climate) Transport

**Table 2:** Sociodemographic and job characteristic of MOs (*n* = 276)

Variable		*n* (%)	Median (IQR)
Age (years old)			30 (3)
Gender	Male	115 (41.7)	
	Female	161 (58.3)	
Race	Malay	54 (19.6)	
	Chinese	175 (63.4)	
	Others	47 (17.0)	
Religion	Islam	55 (19.9)	
	Christian	95 (34.4)	
	Buddhist	80 (29.0)	
	Hinduism	24 (8.7)	
	None	22 (8.0)	
Marital status	Single	182 (65.9)	
	Married	94 (34.1)	
Number of children	0	224 (81.2)	
	More than 1	52 (18.8)	
Medical/Psychiatric illness	No	255 (92.4)	
	Yes	21 (7.6)	
Duration of service (years)			3.5 (2.5)
Department	Internal medicine	40 (14.5)	
	Accident and Emergency	32 (11.6)	
	Anaesthesiology	29 (10.5)	
	Paediatric	20 (7.2)	
	Psychiatry	33 (12)	
	Dermatology	5 (1.8)	
	Surgery	14 (5.1)	
	Obstetrics and Gynaecology	23 (8.3)	
	Orthopaedic	4 (1.4)	
	Otorhinolaryngology	9 (3.3)	
	Ophthalmology	13 (4.7)	
	Dentistry	7 (2.5)	
	Radiology	9 (3.3)	
	Radiotherapy, oncology and palliative care	13 (4.7)	
	Pathology, transfusion medicine, forensic	14 (5.1)	
	Administration and clinical research centre	9 (3.3)	
	Rehabilitative medicine	2 (0.7)	
Postgraduate studies	No	133 (48.2)	
	Yes	143 (51.8)	
Employment status	Permanent	273 (98.9)	
	Contract	3 (1.1)	
Types of working hours/ on-calls	Office hour	22 (8.0)	
	Active	104 (37.7)	
	Passive	23 (8.3)	
	Active and passive	95 (34.4)	
	Shift system	32 (11.6)	

**Table 3:** Factors associated with general poor quality of life in medical officers (*n* = 276)

Variables		*b*	Adjusted OR (95% CI)	*P*-value^a^
Types of working hours/on-calls	Office hour^b^			
	Active	1.41	4.11 (0.98, 17.20)	0.053
	Passive	0.38	1.46 (0.37, 7.99)	0.662
	Active and passive	1.68	5.36 (1.21, 23.79)	0.027*
	Shift system	0.86	2.37 (0.48, 11.65)	0.289
Effort-reward imbalance	Medium effort reward^b^			
	Low effort high reward	−2.19	0.11 (0.05, 0.57)	< 0.001*
	High effort high reward	−0.61	0.54 (0.17, 1.78)	0.314
	Low effort low reward	−0.98	0.37 (0.11, 1.23)	0.110
	High effort low reward	1.55	4.71 (1.71, 13.01)	0.003*
Overcommitment	Medium^b^			
	Low	−0.26	0.77 (0.34, 1.78)	0.546
	High	0.18	1.19 (0.55, 2.57)	0.655

Notes:

a Likelihood ratio test;

b the reference category;

* *P*-value less than 0.05 consider significant;

OR = odds ratio; CI = confidence interval

**Table 4:** Factors associated with poor quality of life in physical and psychological domains in medical officers (*n* = 276)

Variables		Physical domainPhysical domain			Psychological domainPsychological domain		

		*b*	OR (95% CI)	*P*-value^a^	*b*	AOR (95% CI)	*P*-value^a^
Medical/psychiatric illness	No^b^						
	Yes	3.15	23.35 (4.25, 128.45)	0.000*	0.96	2.60 (0.74, 9.10)	0.135
Types of working hours/on-calls	Office hour^b^						
	Active	2.69	14.75 (1.16, 188.35)	0.038*	−0.57	0.57 (0.12, 2.63)	0.470
	Passive	2.76	15.76 (0.86, 289.10)	0.063	0.66	1.93 (0.33, 11.53)	0.469
	Active and passive	2.90	18.25 (1.39, 238.98)	0.027*	−0.36	0.96 (0.21, 4.42)	0.963
	Shift system	2.55	12.8 (0.92, 179.97)	0.058	−0.54	0.59 (0.11, 3.21)	0.537
ERI	Medium effort reward^b^						
	LEHR	−2.57	0.08 (0.01, 0.47)	0.005*	−2.54	0.08 (0.02, 0.38)	0.002*
	HEHR	−1.60	0.20 (0.02, 1.93)	0.166	−1.74	0.18 (0.02, 1.48)	0.110
	LELR	0.96	2.6 (0.63, 10.8)	0.188	−0.08	0.92 (0.24, 3.50)	0.906
	HELR	1.51	4.53 (2.02, 10.17)	0.000*	1.78	5.95 (2.82, 12.58)	< 0.001*
Overcommitment	Medium^b^						
	Low	−0.66	0.52 (0.16, 1.69)	0.276	−0.22	0.80 (0.29, 2.21)	0.665
	High	0.69	1.99 (0.88, 4.52)	0.100	0.46	1.58 (0.73, 3.42)	0.242

Notes:

a Likelihood ratio test;

b the reference category;

* *P*-value less than 0.05 consider significant;

OR = odds ratio; CI = confidence interval

**Table 5:** Factors associated with poor quality of life in social and environmental domain in MOs (*n* = 276)

Variables		Social domain			Environmental domain		

		*b*	OR (95% CI)	*P*-value^a^	*b*	AOR (95% CI)	*P*-value^a^
Gender	Female^b^						
	Male	0.36	1.43 (0.76, 2.69)	0.270	0.71	2.03 (1.04, 3.98)	0.038*
ERI	Medium effort reward^b^						
	LEHR	−2.02	0.13 (0.04, 0.44)	0.001*	−3.31	0.04 (0.01, 0.30)	0.002*
	HEHR	−1.36	0.26 (0.05, 1.34)	0.106	−20.23	0 (0.00, 0.00)	0.998
	LELR	−0.65	0.52 (0.13, 2.12)	0.365	0.14	1.15 (0.31, 4.24)	0.838
	HELR	0.33	1.39 (0.66, 2.90)	0.385	1.44	4.21 (1.95, 9.08)	< 0.001*
Overcommitment	Medium^b^						
	Low	−0.82	0.44 (0.17, 1.16)	0.097	−0.49	0.61 (0.23, 1.65)	0.331
	High	0.38	1.46 (0.71, 2.99)	0.304	−0.38	0.68 (0.31, 1.51)	0.348

Notes:

a Likelihood ratio test;

b the reference category;

* *P*-value less than 0.05 consider significant;

OR = odds ratio; CI = confidence interval
